# Influence of sows’ parity on performance and humoral immune response of the offspring

**DOI:** 10.1186/s40813-018-0111-8

**Published:** 2019-02-14

**Authors:** Carlos Piñeiro, Alberto Manso, Edgar G. Manzanilla, Joaquin Morales

**Affiliations:** 1 PigCHAMP Pro Europa S.L., c. Dámaso Alonso, 14, Segovia, Spain; 2 Teagasc, Pig Development Department, Moorepark, Fermoy, Cork, Ireland; 3 School of Veterinary Medicine, University College Dublin, Belfield, D04 V1W8, Dublin 4, Ireland

**Keywords:** Acute phase protein, Fattening pigs, Immunoglobulin, Multiparous sow, Primiparous sow

## Abstract

**Background:**

Primiparous sows (PP) have higher nutrient requirements, fewer piglets born with lower birth weight and growth performance than multiparous sows (MP). The aim of the current study was to investigate the effect of parity of sow (PP or MP) on the growth performance and humoral immune response of piglets. A total of 10 PP and 10 MP (3rd to 5th parity) sows were used. There were 4 treatments in a 2 × 2 factorial arrangement, with piglets from PP sows suckled by PP or MP sows, and piglets from MP sows suckled by PP or MP sows. Average daily gain (ADG) of piglets during the lactation period, and ADG, average daily feed intake (ADFI) and gain:feed ratio (G:F) from weaning to 144 days of age were controlled, and concentrations of immunoglobulins G (IgG) and major acute phase protein (Pig-MAP) were measured as markers of humoral immune response throughout the study.

**Results:**

Total ADG was higher in piglets born from MP than in those born from PP (669 vs. 605 g/day; standard error of the mean (SEM) = 15.5, *n* = 5; *P* = 0.001) and in piglets suckled by MP than in piglets suckled by PP (655 vs. 620 g/day; SEM = 15.5, *n* = 5, *P* = 0.037). Total ADFI was higher for pigs born from MP than for those born from PP (1592 vs. 1438 g/d, SEM = 42.2, n = 5, *P* < 0.001). Total G:F tended to be higher for pigs suckled by MP than for those suckled by PP (0.43 vs. 0.41, SEM = 0.006, *n* = 5, *P* = 0.076). At weaning, IgG serum concentration was higher (30.0 vs. 17.8 mg/mL, SEM = 4.98, *n* = 15, *P* = 0.013) in pigs suckled by MP than in piglets suckled by PP. However, IgG concentrations were higher for pigs born from PP than for pigs born from MP on days 116 (*P* < 0.001) and 144 (*P* = 0.088). Pig-MAP tended to be lower in pigs suckled by MP than in pigs suckled by PP on days 40 and 60 of age (*P* < 0.10).

**Conclusions:**

The research indicates that the growth performance and humoral immune response of the offspring of PP is improved by cross-fostering with MP. These results open the possibility of an interesting strategy for improving the growth of litters from PP, that is easier to apply than current programs based on parity segregation, which implies a separate building site to house gilts, first parity sows and their offspring.

## Background

Primiparous sows (PP) have higher nutrient requirements [1, 2] and fewer piglets born than multiparous sows (MP) [3]. Usually, PP sows are bred before they reach mature body size and when the back fat levels are still limited and often times below the recommendation of 18.0–23.0 mm at first insemination [4]. Part of the nutrient intake of a PP sow during the reproductive cycle is still used for their own tissue growth [5]. Piglet birth weight and growth performance during lactation are also lower for piglets born to PP sows than to MP sows [6, 7], and these differences may affect piglet growth during the nursery and finishing phases. However, the impact of sow parity on pig growth during the finisher pigs’ whole life span has not been evaluated.

Pigs are born with limited reserves of fat and stored glycogen [8–10]. Thus, an adequate colostrum intake by the newborn pig is key for its survival, providing nutrients for growth and development [11]. Ferrari et al. [12] have recently shown that a minimum of 200–250 g of colostrum is necessary to avoid growth retardation. Furthermore, Decaluwé et al. [13] have shown an association between colostrum intake and growth and survival of the piglet. Colostrum also provides passive immunity derived from maternal immunoglobulin transmission [14]. In pigs, placental transmission of Ig from dam to fetal circulation is not possible [15], and so Ig uptake from colostrum is very important for the protection of the new-born pig. Colostrum and milk also contain high amounts of bioactive components (e.g. insulin-like growth factors, epidermal growth factor, lactoferrin, leptin, nucleotides) which play a role in organ maturation, growth, and disease resistance [16–19]. Because milk composition is linked to mammary development [20] components of colostrum and milk such as Ig, total fat content, or energy and growth factors differ between PP sows and MP sows [7, 21]. Primiparous sows might not be well adapted to the new environment, including acquisition of specific immunity against pathogens present on the farm. Previous studies have reported lower Ig concentrations in blood from PP sows than from MP sows [21–24]. Moreover, the concentration of Ig in neonatal pig serum has been found to be directly proportional to the concentration found in colostrum and in sow serum [22, 24–26]. However, there is some controversy about the effect of colostrum IgG concentration on piglets. Some studies have not observed any effect of IgG concentration in colostrum nor IgG colostrum intake on the health and performance of the litter [27]. However, other studies have found a positive effect of colostrum Ig and milk concentration intake on piglet health [26, 28].

Before specific immune response take place, pigs respond to threats, such as infectious diseases, stress, injuries or traumas, with a series of early-defense system known as acute phase response. Although non-specific, it serves as a core of the innate immune response that contributes to resolution and the healing process, but also induces profound metabolic alterations such as anorexia and increased muscle catabolism [29]. When a disease occurs, concentrations of acute phase proteins (APP) increase and can be used as a non-specific marker of the health status of the animal [30]. The current study investigated the effect of the sow parity (PP or MP) on the growth performance of piglets from birth to slaughter. In addition, serum concentration of major acute phase protein (Pig-MAP) and Ig of piglets were evaluated as markers of humoral immune response from weaning to slaughter and Ig transmission to the piglets, respectively.

## Methods

### Animals husbandry diets and experimental design

A total of twenty sows (Large White x Landrace) at 107 ± 1 d of gestation, comprising 10 PP sows and 10 MP sows (from third to fifth parity) with good health status, were selected. Second parity sows and ‘old-sows’, with more than 5 parities, were discarded and not included in the study because they might show different characteristics and not be representative of MP sows. The experiment was carried out in a commercial farm, positive for porcine reproductive and respiratory disease virus (PRRSV), swine influenza virus (SIV) and *Mycoplasma hyopneumoniae*, and all sows had the same adaptation program. Briefly, normal management of gilts in the farm included arrival of gilts on the farm at 120 d of age, their immunization against the main pathogens that they might be exposed to in the feces of reproductive sows, and their vaccination against major pathogens (Parvovirus, *Erysipelothrix rhusiopathiae*, *Bordetella bronchiseptica* & *Pasteurella multocida* (Atrophic rhinitis), *Mycoplasma hyopneumoniae*, Aujeszky’s disease virus, SIV, and PRRSV) according to standard protocols. At the second estrus the gilts were moved to the gestation barn. Once there, 21 ± 1 d later and coinciding with their third estrus, the gilts were inseminated at the same time that MP sows which were weaned the previous week. At 107 ± 1 d of gestation, the experimental sows were randomly housed in an environmentally controlled farrowing room. This room contained 20 pens (2.2 × 3.0 m) with plastic-slatted flooring and kept in farrowing crates (1.0 × 2.5 m) equipped with a trough feeder with a sow nipple drinker. The pens also had a piglet nipple drinker. Barn temperature was maintained at 23 °C, and supplementary heat was supplied to the piglets for the first week of lactation keeping the temperature at piglet height at 30 °C. During the experiment, animals were checked daily by a veterinarian including the status of the udders.

### Diets

During gestation, sows were fed twice a day with approximately 1.5 kg of a cereal-soybean meal-based diet, formulated to contain 2045 kcal NE/kg, 14.0% crude protein, 0.60% lysine, and 5.4% crude fiber content. During lactation, sows were fed a diet based on the same ingredients, but that contained 2150 kcal NE/kg, 17.0% crude protein, 0.90% lysine, and 4.3% crude fiber content. Both gestation and lactation diets were dry feeds and mixed with water in the feeder. For the first 7 d after farrowing, sows were offered increasing amounts of feed until they reached their ad libitum feed intake. Individual daily feed intake of sows was calculated by subtracting the daily amount of feed supplied from the amount of feed remaining in the feeders at the end of feeding.

### Experimental design

Piglets were ear tagged, weighed and allotted to treatment immediately after birth, before first colostrum intake. The experiment followed a completely randomized design with four treatments organized as a 2 × 2 factorial arrangement, with the parity of sows at farrowing and parity of suckled sow as main factors. Half of the piglets born to a PP sow were also suckled by a PP sow, while the other half were suckled by a MP sow. Equally, half of the piglets born to a MP sow were suckled by the same parity sow, while the other half were suckled by a PP sow. All cross-fostering required were done immediately after birth and before first colostrum intake. Litters were balanced to ensure 10 and 11 piglets for PP sows and MP sows respectively.

The experiment was divided in 3 periods; lactation (from birth to weaning at 28 ± 2 d of age), nursery (from weaning to 74 ± 2 d of age) and growing-finishing (from nursery to 144 ± 2 d of age). After weaning, piglets were allocated by previous treatments and sex to 20 pens of 10 pigs each (5 pens per treatment; 5 entire males and 5 females in each pen). Ten animals were removed randomly from pigs suckled from MP sows to balance the pen density. Piglets were housed in an environmentally controlled wean-to-finish barn. The temperature was set at 28 °C for the first week after weaning and was then decreased by 1 °C per week until reaching 20 °C at 74 d of age, after which it was kept constant. Pens (2.5 × 3.0 m) were provided with plastic-slatted floors, except for a non-slatted central area (1 m wide). Pens were equipped with floor heating, and each pen was provided with 60 cm of through with 3 holes and a nipple drinker. Pigs had ad libitum access to water and pelleted diet throughout the trial. The feeding program was the same for all the pigs, and consisted of 4 diets based on barley, corn, wheat and soybean meal (Table 1) supplied respectively from 28 to 38 d of age (prestarter diet), 38 to 74 d (starter diet), 74 to 116 d (growing diet), and 116 to 144 d of age (finishing diet). All diets met or exceeded the nutritional requirements of the pigs [31]. Animals remained in the same pens from weaning until the end of the experimental period (144 days of age).

**Table 1:** Ingredient composition and calculated nutrient composition of the experimental diets (% dry matter unless indicated otherwise)

	Prestarter (28–38 d)	Starter (38–74 d)	Growing (74–116 d)	Finishing (116–144 d)
Ingredients				
Corn	450.0	450.0	84.0	87.7
Sweet dried milk whey	150.0	50.0	–	–
Extruded soybean	114.0	30.0	–	–
Barley	110.0	220.0	250.0	300.0
Soybean meal 44% CP	100.0	186.0	188.7	93.7
Wheat	–	–	290.0	297.0
Corn DDGS	–	–	100.0	150.0
Glycerin, 85%	–	–	30.0	30.0
Sugar beet molasses	–	–	15.0	15.0
Soybean oil	39.7	24.5	15.7	–
Dicalcium phosphate	16.9	18.7	–	–
Mono-calcium phosphate	–	–	4.0	2.0
Calcium carbonate	0.7	2.5	11.7	13.3
Sodium chloride	2.0	3.1	2.3	2.3
L-Lysine HCl, 78%	5.7	5.1	3.7	4.3
L-Threonine	2.0	2.0	1.1	1.2
Methionine-OH, 88%	2.9	2.3	0.8	0.5
L-Tryptophan	1.1	0.9	–	–
Vitamin-mineral mix^a^	5.0	5.0	3.0	3.0
Nutrients content				
NE, MJ/kg	10.79	10.26	9.83	9.61
CP	191	170	173	150
Digestible Lys	13.5	11.5	9.4	8.0

^a^Vitamin/mineral mix prestarter and starter diets provided the following quantities per kilogram: vitamin A, 10,000 IU; vitamin D_3_, 2000 IU; vitamin E, 100 IU; vitamin K_3_, 2 mg; thiamine, 4 mg; riboflavine, 10 mg; vitamin B_6_, 5 mg; vitamin B_12_, 0.03 mg; biotin, 0.4 mg; niacin, 50 mg; choline, 300 mg; Ca-d-pantothenic acid, 30 mg; folic acid, 1.8 mg; Fe, 60 mg; Zn, 100 mg; Cu, 10 mg; Mn, 50 mg; I, 0.6 mg; Se, 0.2 mg. Vitamin/mineral mixes for growing and finishing diets provided the following quantities per kilogram: vitamin A, 6000 IU; vitamin D_3_, 1500 IU; vitamin E, 20 IU; vitamin K_3_, 2 mg; thiamine, 2 mg; riboflavine, 3 mg; vitamin B_6_, 1 mg; vitamin B_12_, 0.02 mg; niacin, 15 mg; choline, 100 mg; Ca-d-pantothenic acid, 12 mg; Fe, 110 mg; Zn, 110 mg; Cu, 150 mg; Mn, 45 mg; I, 0.8 mg; Se, 0.2 mg

### Data collection

Sow feed intake was controlled individually during the lactation period, and piglets were individually weighed at birth and at 28 ± 2 d of age to calculate the average daily gain (ADG) during lactation. After weaning, feed intake on a pen basis and individual body weight (BW) of the pigs were recorded every 2 weeks. Mortality was daily recorded and weighed. The ADG, average daily feed intake (ADFI), and gain to feed ratio (G:F; based on the calculation ADG:ADFI) were calculated for each period (nursery, growing, and finishing) and for the entire experimental period (28–144 d of age) from these data.

Blood samples (7 mL) were obtained restraining pigs with a snout snare and sampling from the jugular vein. Three piglets per sow were chosen at random (15 piglets per treatment) and were sampled at 14, 28, 38, 60, 90, and 144 d of age using 10 mL vacutainer tubes (BD, San Agustin de Guadalix, Madrid, Spain). Serum was immediately removed after centrifugation at 3500×g for 5 min at room temperature and was kept frozen (− 20 °C) until their analysis for serum concentrations of Pig-MAP and IgG.

### Pig-MAP and IgG determination

Serum Pig-MAP and IgG concentrations were determined by following the methods of Tecles et al. [32] and Broom et al. [33], respectively. Briefly, the concentration of Pig-MAP was determined using a commercial sandwich ELISA kit based on two monoclonal anti Pig-MAP antibodies (Pig-MAP Kit ELISA, PigCHAMP Pro Europa SL, Segovia, Spain), according to the instructions of the manufacturer. Serum IgG quantification was performed using commercial sandwich ELISA kits (Bethyl Laboratories Inc., BioNova, Madrid, Spain).

### Statistical analysis

Statistical analyses were performed as a completely randomized design with 4 treatments arranged as a 2 × 2 factorial arrangement, using the pen as the experimental unit for growth performance as well as the animal for Pig-MAP and IgG concentrations in serum. Power calculations (G*Power, Universität Düsseldorf, Germany) were carried out using data on variability and effect size from previous trials in the same farm adapted to the described arrangement and with growth as primary outcome. Normality of data and models was tested by graphic (P-P, Q-Q plots) and numerical (Shapiro-Wilk test) methods. Data on growth performance were analyzed by repeated measures, with parity of gestating sow and parity of lactating sow, time point, and their interactions as main effects, by using the MIXED procedure of SAS 9.2 (SAS Inst. Inc., Cary, NC). When the gestating and lactating sow parity had a significant interaction with time, *p*-values for each time point were obtained. Data on Pig-MAP and IgG concentrations in serum were analyzed by 2-way ANOVA at each time point. Mortality data showed lack of normality and therefore data was analyzed using the GLIMMIX procedure of SAS for generalized linear mixed models. Tukey’s correction was used for multiple mean comparisons. Alpha level for determination of significance was 0.05 and trends were discussed using an alpha level of 0.10.

## Results

During the lactation period, no clinical signs were observed in the experimental sows and no treatments were required. The average size of the litter at farrowing was of 11.9 ± 2.46 piglets total born and 11.1 ± 2.35 piglets born alive, with no differences detected between MP sows and PP sows, respectively. Also, there was no difference between treatments in the BW of the pigs at birth (1.7 ± 0.05 kg BW) and at weaning at 28 d of life (7.9 ± 0.25 kg BW). During the second week of lactation, MP sows tended to have higher ADFI than PP sows (6.08 vs. 4.99 kg/d, standard error of the mean (SEM) = 0.445, *P* = 0.090). Animals born to MP sows tended to have higher pre-weaning mortality than pigs born to PP sows (5.0 vs. 1.1%; *P* = 0.077). No more than 1 piglet died per litter in any case.

No interactions were found between parity of the gestating and lactating sow for any of the variables studied (*P* > 0.2 in all cases), and therefore the results are presented as main effects and changes with time (Table 2). However, there was an interaction (*P* = 0.002) between the parity groups of gestating sows and time for BW. Likewise, a trend (*P* = 0.069) to the interaction was found between parity of lactating sow with time for BW. Parity of the gestating or lactating sow did not affect BW of piglets at weaning. However, pigs born to MP sows showed higher BW than those born to PP sows at 116 d (62.4 vs. 57.3 kg, SEM = 1.84, *P* = 0.015) and 144 d (87.0 vs. 79.4 kg, SEM = 2.15, *P* = 0.002) of age. Also, pigs suckled by MP sows showed higher BW than piglets suckled by PP sows at 76 d (32.6 vs. 30.2 kg, SEM = 1.29, *P* = 0.083), 116 d (61.7 vs. 58.0 kg, SEM = 1.84, *P* = 0.065) and 144 d (85.4 vs. 80.9 kg, SEM = 2.15, *P* = 0.052) of age. Total ADG was higher in piglets born to MP sows than in piglets born to PP sows (669 vs. 605 g/d; SEM = 15.5, *P* = 0.001). Also, piglets suckled by MP sows showed higher ADG than piglets suckled by PP sows (655 vs. 620 g/d, SEM = 15.5, *P* = 0.037). Cumulative ADFI was only affected by the parity of the gestating sow. ADFI was higher for pigs born to MP sows than for pigs to PP sows (1592 vs. 1438 g/d, SEM = 42.2, *P* < 0.001). Also cumulative G:F tended to be higher for pigs suckled by MP sows than for those suckled by PP sows (0.43 vs. 0.41, SEM = 0.006, *P* = 0.076). Parity of lactating sow affected mortality between 28 and 144 d, being lower for pigs suckled by MP sows than for pigs suckled by PP sows (4.0 vs. 10.3%, *P* = 0.035).

**Table 2:** Growth performance of pigs from weaning to slaughter^1^:28 to 144 d of age

Gestation	Multiparous (MP)		Primiparous (PP)		SEM^2^	*P*-value^3^	
Lactation	MP	PP	MP	PP	(*n* = 5)	Gestation	Lactation
Body weight, kg							
28 d	8.4	7.9	7.9	7.7	0.53	0.512	0.454
74 d	33.5	30.7	31.6	29.6	1.29	0.273	0.083
116 d	63.5	61.2	59.8	54.8	1.84	0.015	0.065
144 d	88.0	86.0	82.7	75.7	2.15	0.002	0.052
Average daily gain, g							
28–144 d	675	663	634	576	15.5	< 0.001	0.037
28–74 d	524	476	494	457			
74–116 d	750	760	707	625			
116–144 d	816	816	763	712			
Average daily feed intake, g							
28–144 d	1602	1582	1474	1401	42.0	< 0.001	0.358
28–74 d	799	746	773	747			
74–116 d	2004	2033	1854	1770			
116–144 d	2386	2381	2120	2005			
Gain to feed ratio							
28–144 d	0.42	0.42	0.43	0.41	0.006	0.526	0.076
28–74 d	0.66	0.64	0.64	0.61			
74–116 d	0.37	0.37	0.38	0.35			
116–144 d	0.34	0.34	0.36	0.36			

^1^Gestation indicates the type of sow from which piglets were born, and lactation type indicates the type of sow which suckled the piglets

^3^SEM: standard error of the mean

^2^All variables were analyzed by repeated measures. The model included type of gestating (G) and lactating (L) sow, age of the piglet (A), and their interactions GxL, GxA, LxA, and GxLxA. Age was always significant (*P* < 0.001), while other interactions were not significant for any variable (*P* > 0.10) except for body weight. The *P* values of the interactions (GxA, LxA) for body weight were 0.001 and 0.069, respectively

An interaction trend (*P* = 0.083) was found between the gestating and lactating sow parity effects for Pig-MAP at 14 d of age; for pigs born to MP sows, those suckled by MP sows tended to have lower serum Pig-MAP concentrations than those suckled by PP sows (0.45 vs. 0.77 mg/mL, SEM = 0.093, Table 3). After weaning, Pig-MAP tended to be lower in pigs that were suckled by MP sows than in pigs suckled by PP sows (0.74 vs. 1.01 mg/mL, SEM = 0.146, *P* = 0.070 at 40 d of age and 0.63 vs. 0.80 mg/mL, SEM = 0.101, *P* = 0.089 at 60 d of age). Also, at 116 d of age, an interaction trend (*P* = 0.098) was detected between gestating and lactating sow; for pigs born to PP sows, those suckled by MP sows had lower levels of Pig-MAP in serum than those suckled by PP sows (0.51 vs. 1.14 mg/mL, SEM = 0.182).

**Table 3:** Major acute phase protein of pigs (Pig-MAP) serum concentration in pigs during lactation, nursery, and growing-finishing phases, mg/mL^1^

Gestation	Multiparous (MP)		Primiparous (PP)		SEM^2^	*P*-value		
Lactation	MP	PP	MP	PP	(*n* = 15)	Gestation	Lactation	Interaction
d of age								
14	0.45	0.77	0.77	0.76	0.093	0.095	0.096	0.083
28	0.93	1.12	0.80	0.77	0.170	0.174	0.646	0.513
40	0.74	1.12	0.75	0.91	0.146	0.500	0.070	0.455
60	0.58	0.88	0.69	0.73	0.101	0.837	0.089	0.210
90	1.02	0.88	0.72	1.17	0.198	0.960	0.432	0.137
116	0.50	0.52	0.51	1.14	0.182	0.088	0.077	0.098
144	0.59	0.64	0.79	0.68	0.082	0.143	0.4	0.309

^1^Gestation indicates the type of sow piglets were born from and lactation indicates the type of sow which suckled the piglets, defined as multiparous sows (from 3 to 5 parities) and primiparous sows

^2^SEM: standard error of the mean

At 28 d of age, IgG concentration was higher in pigs suckled by MP sows than in pigs suckled by PP sows (30.0 vs. 17.8 mg/mL, SEM = 4.98, *P* = 0.013; Table 4). At 40 d of age, pigs born to MP sows tended to have higher levels of IgG than pigs born to PP sows (15.4 vs. 7.4 mg/mL, SEM = 4.25, *P* = 0.084), and pigs suckled by MP sows tended to have higher levels of IgG than pigs suckled by PP sows (15.8 vs. 7.8 mg/mL, SEM = 4.25, *P* = 0.052). However, at 60 d of age, pigs suckled by MP sows had lower IgG concentration compared to pigs suckled by PP sows (4.2 vs. 6.7 mg/mL, SEM = 0.95, *P* = 0.010). Also, pigs born to MP sows had lower IgG concentrations in serum at 116 d of age than pigs born to PP sows (17.0 vs. 30.9 mg/mL, SEM = 3.68, *P* < 0.001), and concentrations still tended to be lower at 144 d of age (35.5 vs. 45.0 mg/mL, SEM = 6.24, *P* = 0.088).

**Table 4:** Immunoglobulin G (IgG) serum concentration in pigs during lactation, nursery, and growing-finishing phases, mg/mL^1^

Gestation	Multiparous (MP)		Primiparous (PP)		SEM^2^	*P*-value		
Lactation	MP	PP	MP	PP	(*n* = 15)	Gestation	Lactation	Interaction
d of age								
14	33.4	34.4	29.8	40.8	7.15	0.847	0.406	0.487
28	23.7	18.9	37.0	16.4	4.98	0.282	0.013	0.119
40	23.0	7.7	8.6	7.0	4.25	0.084	0.052	0.113
60	5.0	6.9	3.4	6.6	0.95	0.300	0.010	0.495
90	21.1	26.6	17.3	20.4	3.68	0.175	0.242	0.736
116	19.5	13.9	30.8	30.9	3.68	0.001	0.451	0.445
144	34.8	36.4	49.0	43.5	6.24	0.088	0.751	0.564

^1^Gestation indicates the type of sow piglets were born from and lactation indicates the type of sow which suckled the piglets, defined as multiparous sows (from 3 to 5 parities) and primiparous sows

^2^SEM: standard error of the mean

## Discussion

Results from the current study demonstrate that both the gestating sow parity and lactating sow parity can affect pig growth performance throughout its productive life, resulting in important differences by the time pigs reach market weight. Pigs born to PP sows tend to be less viable and to have lower growth rates than those born to MP sows [7, 12]. The reason for these differences is a subject of debate and may be related to innate factors of the piglets born to PP sows, such as fewer muscle fibers, or to lower total production [34] and immunoglobulin composition [7] of colostrum and milk of PP sows compared with MP sows. This observation might be important under practical conditions, because growth performance of litters from PP sows could be improved by cross-fostering with MP sows, or through nutritional changes in the post-weaning phase such as increasing threonine and tryptophan content, both involved in biological functions such as gut integrity and immunity [35–37], or supplementing feed with a source of dietary fiber [38]. In the current experiment, final BW (at 144 days of age) was 9% lower for pigs born to PP sows than for pigs born to MP sows, and 7% lower for litters suckled by PP sows than for litters suckled by MP sows. Consequently, fostering extra pigs produced by PP sows with MP sows might mitigate BW growth delay to some extent, with effects that are apparent during the whole productive life.

The reproductive cycle and hormonal system of PP sows are naïve, and their development is still competing for nutrients with their muscle development, while they show lower feed intake capacity and lower metabolizable fat and protein stores [1]. In contrast, mature MP sows are fully-grown and have a well-established reproductive cycle [39]. Under these circumstances, fetal nutrient supply might differ between PP sows and MP sows, thus affecting the fetal development. Averette et al. [40] and Moore [41] have reported a lower birth weight for pigs born to PP sows compared to pigs born to MP sows, indicating that this difference could be due to retardation in fetal growth as well as to fewer skeletal muscle fibers, which cannot be compensated for during the postnatal growth [42], even when no differences in BW are detected at birth [43] as happened in the present study.

The differences in growth observed between piglets from PP sows and MP sows might be explained by transmission of pathogens from the dam to the fetus [44] or due differences in prenatal stress [45, 46]. In our study, Pig-MAP concentration was increased after weaning in pigs suckled by PP sows as compared to pigs suckled by MP sows, which might be indicative of higher exposure to infection or inflammation suffered at weaning by these PP sow suckled pigs.

A reduced Ig concentration during lactation may result in reduced health status and poorer growth performance of nursery pigs [12]. Values obtained in our trial showed a difference of 44% in IgG concentrations in piglets at weaning between those suckled by PP sows and MP sows. Pigs suckled by MP sows showed better growth rates and lower Pig-MAP concentrations in the nursery period than pigs suckled by PP sows, which may indicate a better health status of this group of animals. However, in the present study pigs were cross-fostered immediately after birth and first colostrum intake. It is important for newborn piglets to consume colostrum from their own dams to get adequate immunity [11]. Colostral cells of sows other than a piglet’s own dam are not absorbed and thus cannot confer cellular immunity on the newborn piglets [47]. The precise role of these immune cells in piglet immunity, which was not evaluated in the present study, is not known. Indeed, it has been stated that cross-fostering procedure management carried out before 6–12 h after birth negatively affects transfer of maternal specific humoral and cell-mediated immunity from sow to piglets [48]. Pig growth immediately after weaning was affected by the parity of the lactating sow. However, the parity of the gestating sow had less effect in this early phase of the growing period, although it did affect growth later. This difference in effects may be due to innate traits in maternal immunity. Maternal immunity provided by the lactating sow to the offspring decayed from IgG levels around 30 mg/mL at 14 d of age to around 6 mg/mL at 60 d of age. Then, the pig developed its own immunity, and serum IgG concentrations in pigs increased again with time to reach levels around 35 mg/mL for pigs born to MP sows and 45 mg/mL in pigs born to PP sows at 144 d of age. There is no obvious reason for the difference in the IgG concentrations reported although it could be related to the reduced growth rate reported for pigs born to PP sows. The higher IgG concentrations in pigs born to PP sows might reflect a higher incidence of disease in this group, a less effective innate immunity, or differences in the regulatory components of the immune system with predominance of the humoral response.

## Conclusion

In conclusion, the research indicates that the growth performance and health status of the offspring of PP sows is improved by cross-fostering with MP sows. These results open the possibility of an interesting strategy for improving the growth of litters from PP sows, a strategy that is easy to apply compared with current programs based on parity segregation. However, it is important to note that the results obtained in the current study might have also been affected by factors such as the health status of the studied herd, as well as the management conditions and feeding programs. Further studies are needed to gain more knowledge on the possibilities of this strategy. Pig-MAP and IgG concentrations in serum help explain some of the differences found in production data.

## Abbreviations

ADFI: Average daily feed intake
ADG: Average daily gain
APP: Acute phase protein
BW: Body weight
G:F: Gain to feed ratio
Ig: Immunoglobulin
MP: Multiparous
Pig-MAP: Major acute phase protein of pig
PP: Primiparous
